# A time-resolved fluoroimmunoassay to assay the rabies virus glycoprotein: application for estimation of human rabies vaccine potency

**DOI:** 10.1038/s41598-017-07687-7

**Published:** 2017-08-04

**Authors:** Guanfeng Lin, Shaolang Chen, Hui Zhao, Junyu Liang, Qiaoting Deng, Rongliang Liang, Xinxin Guo, Zhenhua Chen, Baihong Chen, Tiancai Liu, Yingsong Wu

**Affiliations:** 1 Teaching and Scientific Research Center, School of Laboratory Medicine and Biotechnology, Southern Medical University, Guangzhou, China; 2 Institute of Antibody Engineering, School of Laboratory Medicine and Biotechnology, Southern Medical University, Guangzhou, China; 3 Department of Human Anatomy, School of Basic Medical Sciences, Southern Medical University, Guangzhou, China; 4 Guangdong Provincial Key Laboratory of Tropical Disease Research, School of Public Health, Southern Medical University Guangzhou, Guangzhou, China

## Abstract

Replacement of the *in vivo* rabies vaccine potency test (NIH test) by *in vitro* methods had been discussed by several researcher including WHO expert working groups. In this paper, a time-resolved fluoroimmunoassay (TRFIA) for the assay of rabies virus glycoprotein in rabies vaccine was first established to estimate the rabies vaccine potency by using specific monoclonal antibody that only recognized the native, trimeric and immunogenic form of rabies virus glycoprotein. Potency of the rabies virus glycoprotein was assayed with satisfactory performance under optimal conditions, and the method demonstrated satisfactory results when applied in practical samples. The correlation coefficient of potency values obtained from the present TRFIA and ELISA was 0.912, and 0.903 for those from the present TRFIA and NIH test. These preliminary results confirmed that this TRFIA can replace ELISA with higher performance, and could be a promising replacement of the NIH test. Based upon these results, the present TRFIA seemed to be a convenient tool for evaluating rabies vaccine potency and its products at different stages accordingly.

## Introduction

Rabies is an endemic and fatal zoonotic disease, and causes 55,000 human rabies deaths in more than 150 countries and regions per year^1^. Although significant scientific has been made, rabies remains a serious zoonotic disease globally and continues to present challenges for public health safety. Fortunately, rabies is a preventable disease and vaccination is considered as the most viable and cost-effective method for prevention of it refs 2 and 3. Over 15 million people in the world are receiving multi-dose post-exposure prophylaxis to prevent rabies annually^4^. Safe and efficacious vaccines are needed in prevention and post-exposure therapy.

Vaccine potency testing is necessary to evaluate the immunogenicity of inactivated rabies virus vaccine preparations before application^5^. Currently, the National Institutes of Health (NIH) test is recommended by the WHO expert committee to evaluate potency of rabies virus vaccine. However, NIH test has numerous disadvantages such as poor precision, significant variability, inherent concerning cost, violation of animal welfare and biosafety requirements^6, 7^. As a result, there is increased exposure in human beings to live and virulent rabies strains. The NIH test also requires a secure biosafety level 3 facility for housing and challenging the experimental animals. The replacement of the NIH test for rabies vaccine evaluation by *in vitro* methods had been discussed in several research and also by WHO expert working groups^8^. The viral genome of rabies virus produces five monocistronic mRNAs encoding the nucleoprotein, phosphoprotein, matrix protein, transmembrane glycoprotein and the viral RNA-dependent RNA polymerase^9^. The amount of immunogenic rabies virus glycoprotein decides the vaccine potency in the vaccine preparation^10^, and using specific glycoprotein monoclonal antibody (MAb) to evaluate the rabies vaccine potency has been recognized and applied. Several *in vitro* methods have been proposed for the evaluation of vaccines potency based on rabies virus glycoprotein quality and quantity, which is expected to correlate with vaccine potency^2, 8, 10–17^. However, the method in almost of the previous reports was enzyme-linked immunosorbent assay (ELISA) method or based on the extension of ELISA. Due to the characteristics of enzyme conjugates, limitations of ELISA such as low sensitivity, instability, imprecision, narrow detection range and more time consumption are obvious. Therefore rapid, precise and sensitive *in vitro* detection method is needed for the quality control of rabies vaccine. Using europium (Eu) chelates as the labels, Time-resolved fluoroimmunoassay (TRFIA) was considered as a successful non-isotopic detection method since it was first reported by Lovgren *et al*.^18^, and had been noticed as an excellent performance method and employed in sorts of biomedical sciences fields^19–26^. Time-resolved fluoroimmunoassay gets excellent precision, higher sensitivity and extremely wider detection range relative to traditional ELISA^27^. Application of TRFIA for detecting viral proteins antigen had been proven to be very applicable by us in the past research^23, 24^. For acute infectious diseases such as hepatitis B, TRFIA with high sensitivity can assay low level of viral protein in the early stages for disease diagnosis. Although high sensitivity may be not very important for the detection of rabies virus protein, TRFIA with extremely wider detection range, excellent precision and simple operation can greatly save the operation time and workload with much precise and accurate determinations. Using specific MAbs that only recognized the native, trimeric and immunogenic form of rabies virus glycoprotein prevented detection of non-immunogenic, soluble glycoprotein in vaccines, we herein first designed a novel TRFIA which was designed to estimate the potency of human rabies vaccines by assaying glycoprotein in rabies vaccines, and may have utility in replacement of the NIH test. Thus, the aim of our study was to establish this novel TRFIA and validate its application. This study involved measurement of parameters, such as sensitivity, precision, recovery, linearity and feasibility.

## Materials and Methods

### Virus, cell, animal and Samples

The Challenge Virus Strain (CVS) rabies virus and aG strain rabies virus human vaccine were kindly provided by Guangzhou Promise Biologic Products (Guangzhou, China). Purified glycoprotein was prepared by Guangzhou Promise Biologic Products (Guangzhou, China), which was extracted from the rabies virus grown in Vero cell with mild non-ionic detergents and then purified by isoelectric focusing in a sucrose gradient. Sp2/0 cell was stored in Institute of Antibody Engineering, School of Laboratory Medicine and Biotechnology, Southern Medical University (Guangzhou, China). 6–8 weeks old female, BALB/c mice used in this study were procured from Experimental Animal Center, Southern Medical University (Guangzhou, China). Other rabies samples were kindly provided by Guangzhou Promise Biologics Products (Guangzhou, China) and were stored at −80 °C. This study had been approved and registered by the laboratory animal welfare and ethics committee of Southern Medical University (Guangzhou, China). The care and use of the animals conform to the Institutional Animal Ethics Committee guidelines.

### Reagents and instrumentation

Bovine serum albumin (BSA), Tween-20, triton X-100, β-naphthoyltrifluoroacetate, tri-*n*-octylphosphine oxide and tris were procured from Sigma-Aldrich (St. Louis, MO, USA). Sephadex G-50 was purchased from Amersham Pharmacia Biotech (Piscataway, NJ, USA). The Rabies Virus Glycoprotein ELISA Kit (EIA-2489) was procured from DRG Instruments GmbH (Marburg, Germany). Pierce™ BCA Protein Assay Kit (23225) was procured from Thermo Fisher Scientific (Waltham, MA, USA). Eu^3+^-labeling kit, the Victor^3^ 1420 multilabel counter and 96-well plates were procured from PerkinElmer WALLAC (Turku, Finland). Ultra-pure water obtained using a Milli-Q water purification system (Millipore, MA, USA) was used throughout the experiments. Other chemicals and reagents used were of analytical grade and used without further purification.

### Preparation of monoclonal antibodies

Monoclonal antibodies were prepared using standard protocols by immunizing 6–8 weeks old female BALB/c mice with rabies virus (aG strain)^28^. The spleen cells isolated from those BALB/c mice were fused with Sp2/0 cells to prepare the hybridoma. The hybridoma cells secreting monoclonal antibodies against glycoprotein of rabies were screened by indirect immunofluorescent assay with purified glycoprotein, and the monoclonal hybrids were selected after two rounds of single cell cloning. Paired anti-glycoprotein MAbs (S053 and S036) were selected for further study.

### Solutions

Coating buffer was 50 mM Na_2_CO_3_-NaHCO_3_ buffer (pH 9.6). Blocking solution was 50 mM Na_2_CO_3_-NaHCO_3_ buffer (pH 9.6) containing 1% BSA. Labeling buffer was 50 mM Na_2_CO_3_-NaHCO_3_ (pH 8.5) with 155 mM NaCl. Elution buffer was 50 mM Tris-HCl (pH 7.4) with 0.2% BSA and 0.9% NaCl. Standard buffer was 50 mM Tris-HCl (pH 7.8) containing 0.1% NaN_3_ and 0.2% BSA. Assay buffer was 50 mM Tris-HCl (pH 7.8) with 0.02% BSA, 0.05% Tween-20 and 0.05% NaN_3_. Enhancement solution was 100 mM acetate-phthalate buffer (pH 3.2) containing 15 μM β-naphthoyltrifluoroacetate, 50 μM tri-*n*-octylphosphine oxide and 0.1% triton X-100. Washing buffer was 25 mM Tris-HCl (pH 7.8) with 0.9% NaCl and 0.06% Tween-20.

### Immobilization of MAb

Each well of the standard, bare 96-well plates was coated with 250 ng of anti-glycoprotein MAb (S036) diluted in 100 μl coating buffer and incubated overnight at 4 °C. After three times washing, blocking solution (250 μl) was added to the well for blocking the coated surface for 1 h at 37 °C. Finally, the 96-well plates were dried in a high vacuum, and then stored at −20 °C in a sealed plastic bag with desiccant.

### Labeling of MAb

According to the protocol provided by the Eu^3+^-labeling kit, anti-glycoprotein MAb (S053) was labeled with Eu^3+^-chelates. Briefly, 400 μg of Eu^3+^-chelates was gently mixed with 2 mg MAb S053 in 200 μl labeling buffer and incubated with continuous gently shaking for 18 h at room temperature. Then the mixture was separated by a Sephadex G-50 column. Purified Eu^3+^-MAb conjugates were eluted with a descending elution buffer, and collected (1.0 ml per fraction). The protein concentration of Eu^3+^-MAb conjugates in collected fraction was measured by the Pierce™ BCA Protein Assay Kit. The fluorescence of Eu^3+^-MAb conjugates diluted with enhancement solution (1:100) was detected in microtitration wells (200 μl per well). Fractions from the first peak with the highest Eu^3+^ count were pooled and characterized. After dilution with elution buffer containing 0.2% BSA as a stabilizer, purified conjugate was rapidly lyophilized under high vacuum and stored at −20 °C.

### Preparation of standards

After be extracted and purified as described above, potency value of purified glycoprotein was detected by the ELISA kit (EIA-2489) for preparing standards. Standards in the seven mixed standards were prepared by diluting purified rabies glycoprotein in standard buffer as 0, 31.25, 62.5, 125, 250 and 500 international units per milliliter (IU/ml).

### Immunoassay design

**Figure 1:**
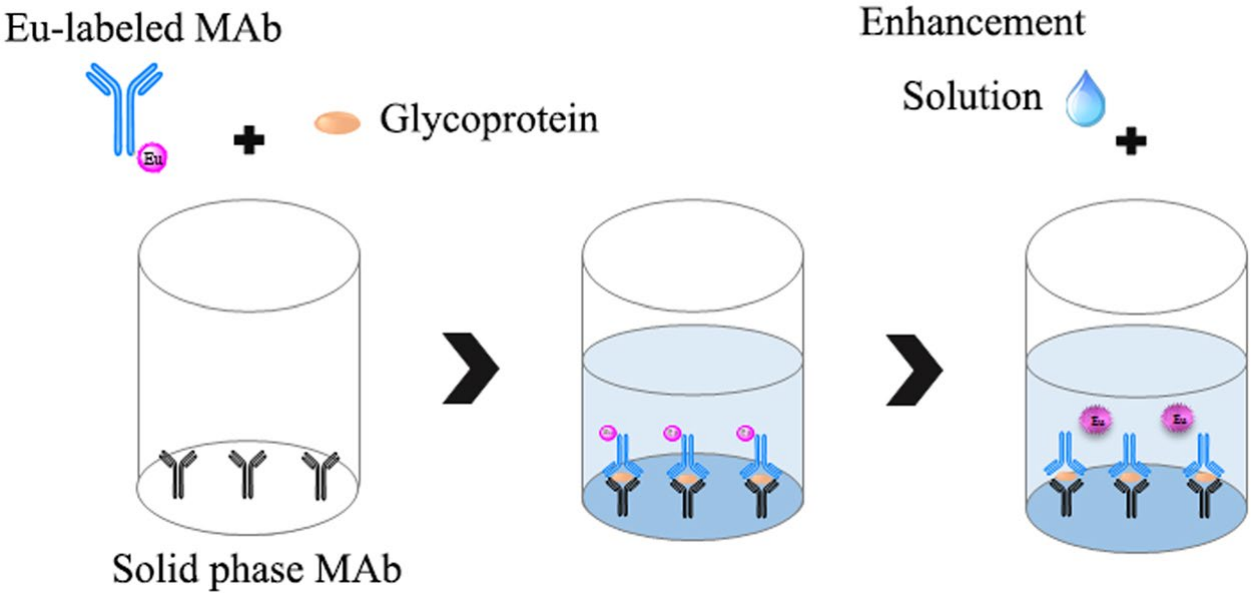
Example of the present TRFIA employing a europium chelate label.

As shown in Fig. 1, the assay was performed using the one-step procedure. Following the immobilization and antibody labeling protocols described above, 50 μl of standards or samples and 100 μl of assay buffer containing 400 ng of Eu^3+^-labeled MAb S053 were added into each well with immobilized MAb S036. After one-hour incubation with continuous slow shaking, immobilized MAb, samples or standards and the Eu^3+^-labeled MAb made up a sandwich-type format in wells. Then, the wells were washed for 6 times and filled with 100 μl of enhancement solution. The plates were shaken for 5 min at room temperature and then the fluorescence intensity was measured on the Victor^3^ 1420 Multilabel Counter equipped with filters for Eu^3+^ (excitation wavelength, 340 nm; emission wavelength, 613 nm; delay time,0.40 ms; window time, 0.40 ms; cycling time, 1.0 ms).

### NIH test

As per the guidelines of the Institutional Animal Care and Use Committee (IACUC), Southern Medical University, for prevention and control rabies, the NIH test was carried out in BALB/c mice following standard procedures^29^. Five-fold serial dilutions of the unknown vaccine and reference vaccine were prepared for immunizing 12–14 g mice by intraperitoneal injection with 0.5 ml dose. Mice were immunized again with the same procedure after one week. The fourteenth day after the first time immunization, intracerebral challenge with lethal dose of CVS strain was performed for the mice. The mice was observed and recorded daily for morbidity and mortality until the twenty-eighth day since the first time immunization. Potency value of the vaccine sample was calculated by using the Reed-Muench method. Thirty nine vaccine samples were tested using the present TRFIA method and the potency of each vaccine sample was re-evaluated using NIH test.

### Statistical analyses

Analysis of data was performed using Statistical Product and Service Solutions (SPSS) software (version 20.0, SPSS Inc., Chicago, IL). Two-tailed test was applied for statistical analysis in all tests with alpha level set at *α* = 0.05. A P value of less than 0.05 (*P* < 0.05) was considered statistically significant. Because the fluorescence signal value is too large, a logarithmically transformed data was employed for curve fitting. Standard curves were obtained by plotting the logarithm of fluorescence intensity (Y) against the logarithm of the sample value (X) and fitted to a four-parameter logistic equation using Origin7.5 SR1 (Microcal, USA): ${\rm{LogY}}={\rm{A}}\times {\rm{LogX}}+{\rm{B}}$.

## Results

### Fitting standard curve for the relationship between concentration and fluorescence signal

**Figure 2:**
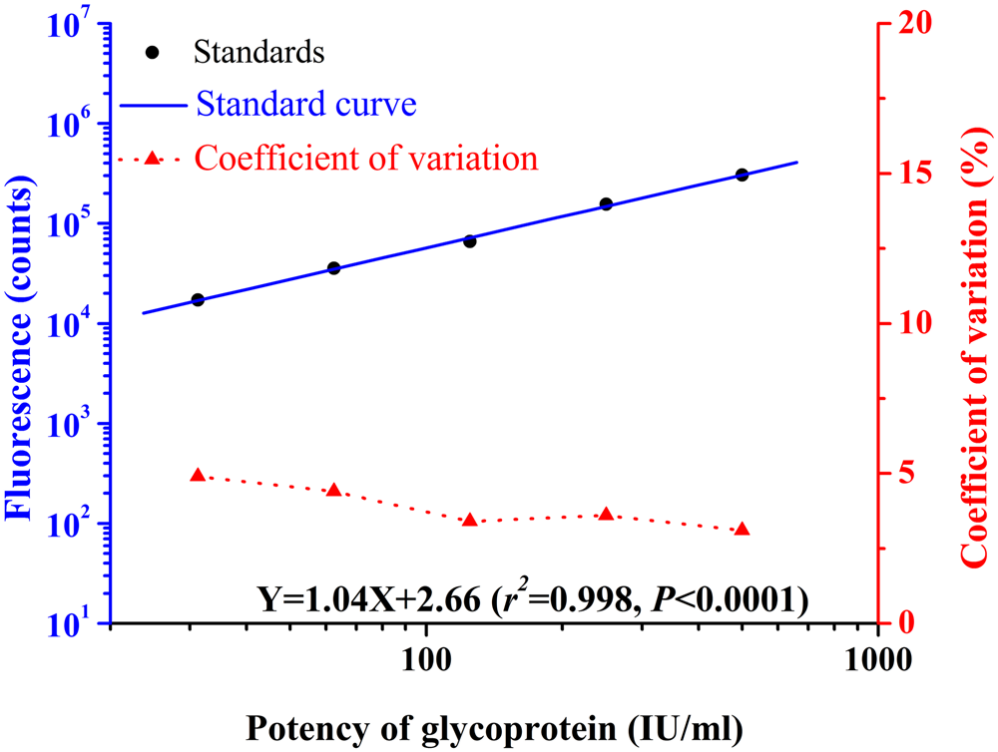
Standard curves and intra-assay precision profile for the present TRFIA (each point was based on 10 replicates).

**Figure 3:**
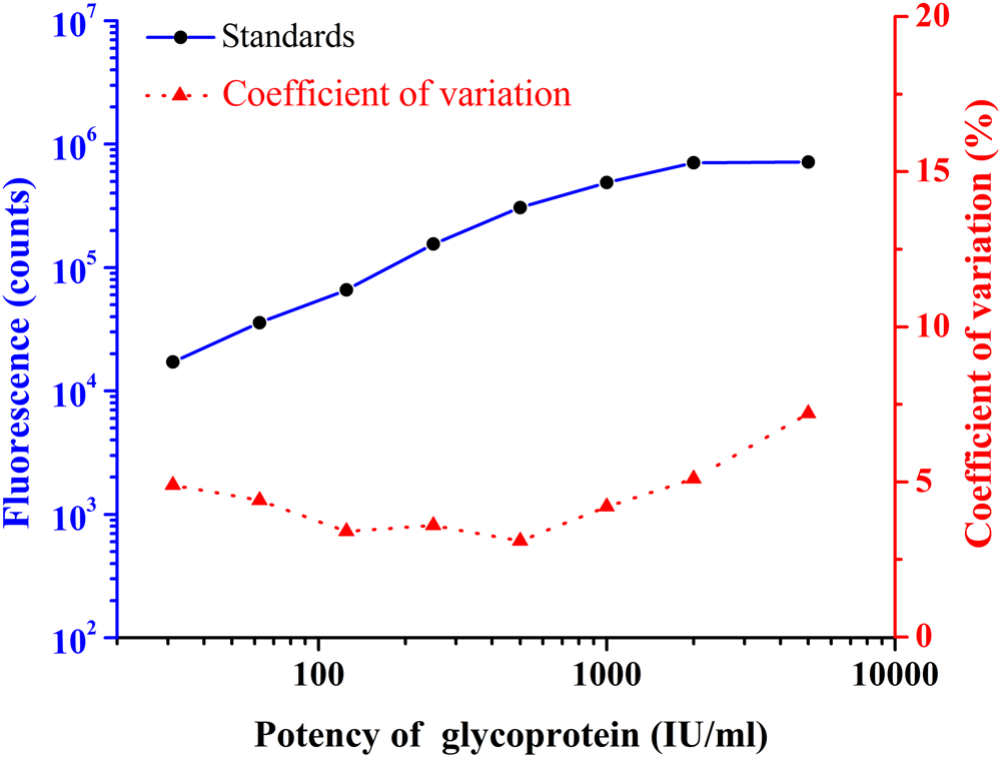
High-dose signal saturation (hook-effect) for the present TRFIA (each point was based on 10 replicates).

Fluorescence signal was carried out following our immunoassay design with a series dilution of standards (0, 31.25, 62.5, 125, 250 and 500 IU/ml) obtained from 10 separate assays. As shown in Fig. 2, Coefficients of variation of each standard were less than 10% for fitting the standard curve. For the standard curve depicted in Fig. 2, the best-fit calibration was determined to be described by the following equation: ${\rm{LogY}}=1.04\times {\rm{LogX}}+2.66$ (*r* ^*2*^ = 0.998, *P* < 0.0001). As fluorescence signal value of the unknown sample is collected by the instrument, the sample will be quantitative determination by using this equation. Signal saturation (hook effect) means falsely low values on an immunoassay when an overwhelming amount of antigen affects the binding capacity of the added antibody, especially when using the double antibody sandwich method. As shown in Fig. 3, signal saturation appeared when the dose exceeded 1 000 IU/ml for the present method. This means that when the dose exceeds 1000 IU/ml, the relationship between concentration and fluorescence signal no longer meet the linear relationship of the fitting equation.

### The minimum detectable dose of the present method for assaying glycoprotein potency

The minimum detectable dose was determined by adding two standard deviations (SD) to the mean (mean + 2*SD) optical density value of twenty zero standard replicates and calculating the corresponding concentration. According to this calculation method, a value of 0.098 IU/ml was identified as the minimum detectable value of the present method for potency assay of glycoprotein (Table 1).

**Table 1:** Repeated measure of zero standard for assaying the minimum detectable dose.

Number of replication	Mean	Standard deviation	Mean + 2*SD	Minimum detectable dose
20	575	23	621	0.098 IU/ml

### Repeatability and recovery test for the present method

The intra-assay and inter-assay precision were analyzed using three samples and the same batch of reagents on separate days as shown in Table 2. Total coefficient of variations of the present assay ranged from 2.9% to 4.9%. As expected, the repeatability of the present assay was remarkably low, neither of imprecision was significant (≥10%). The general analytical recovery of the assay was in the range of 90–110%. Therefore the accuracy of the measurements will be guaranteed by the present method.

**Table 2:** Repeatability and recovery of the present TRFIA.

	Samples	Nominal value (IU/ml)	Mean ± SD (IU/ml)	CV (%)	Recovery (%)
Intra-assay (n = 10)	A	46.1	44.6 ± 1.83	4.1	96.7
	B	70.1	68.9 ± 2.68	3.9	98.2
	C	124.2	126.1 ± 3.65	2.9	101.5
Inter-assay (n = 12)	A	46.1	45.8 ± 2.24	4.9	99.3
	B	70.1	71.3 ± 2.64	3.7	101.7
	C	124.2	128.4 ± 4.36	3.4	103.4

CV: coefficient of variation.

SD: standard deviation.

### Dilution linearity test for the present method

For linearity testing, serial dilutions of the three glycoprotein samples (33.1, 90.1 and 214.2 IU/ml) were prepared and the potency of each dilution was determined by the present method. Table 3 showed the dilution linearity of this assay when we used samples serially diluted with assay buffer, expected values were derived from initial value of potency in the undiluted samples. Correlating the results obtained from assay with the expected values, we found that the expected values were identical with measured values. This result confirmed that the detection would not be affected if the sample was diluted with assay buffer.

**Table 3:** Dilution Linearity test of the present TRFIA.

Sample	Dilution	Value (IU/ml)		
		Expected	Observed (n = 3)	Recovery
A	NA		33.1	
	1:2	16.6	17.1	103.0%
	1:4	8.3	8.1	97.6%
	1:8	4.1	4.0	97.6%
	1:16	2.1	2.2	104.8%
B	NA		90.1	
	1:2	45.1	46.5	103.1%
	1:4	22.6	23.2	102.6%
	1:8	11.3	10.9	96.5%
	1:16	5.7	5.8	101.8%
C	NA		214.2	
	1:2	107.1	106.5	99.4%
	1:4	53.6	54.9	102.4%
	1:8	26.8	27.5	102.6%
	1:16	13.4	12.8	95.5%

NA, not applicable.

### Comparison of assay results and performance for the present TRFIA and ELISA

**Figure 4:**
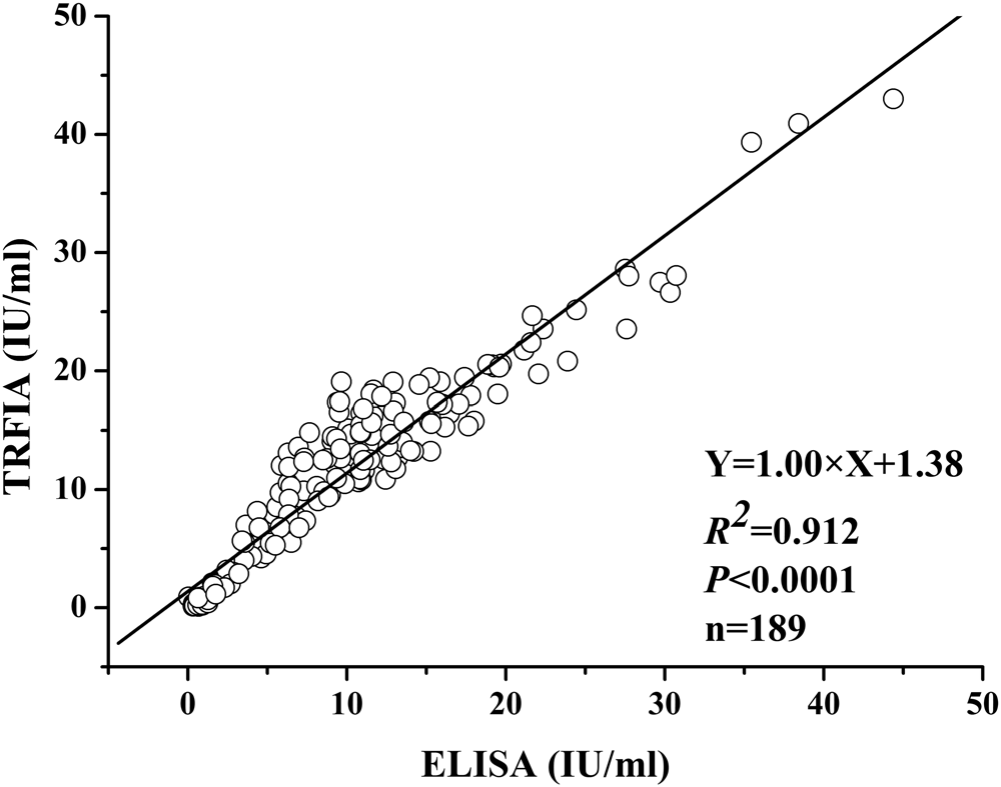
Graphical comparisons of the present TRFIA and ELISA results for assay of rabies virus glycoprotein.

According to the published data in the protocol provided by the Rabies Virus Glycoprotein ELISA Kit (EIA-2489), comparison of assay performance for the present TRFIA and ELISA was shown in in Table 4. Potency values of glycoprotein in 189 samples including rabies samples, semi-finished vaccine samples, unqualified and qualified vaccine samples were assayed by the present TRFIA and ELISA, respectively. As shown in Fig. 4. The correlation coefficient of potency values obtained from the present TRFIA method and ELISA method was excellent, with a regression equation: ${\rm{Y}}=1.00\times {\rm{X}}+1.38$ (*r* ^*2*^ = 0.912, *P* < 0.0001).

**Table 4:** Comparison of assay performance for the present TRFIA and ELISA.

Method	Recovery	Imprecision	Operating time	Maximum quantitative value
TRFIA	96.7–103.4%	2.9–4.9%	1.5 h	1 000 IU/ml
ELISA	80–120%	<20%	3 h	25 IU/ml

### Comparison of potency values obtained from the present TFRIA and NIH test

**Figure 5:**
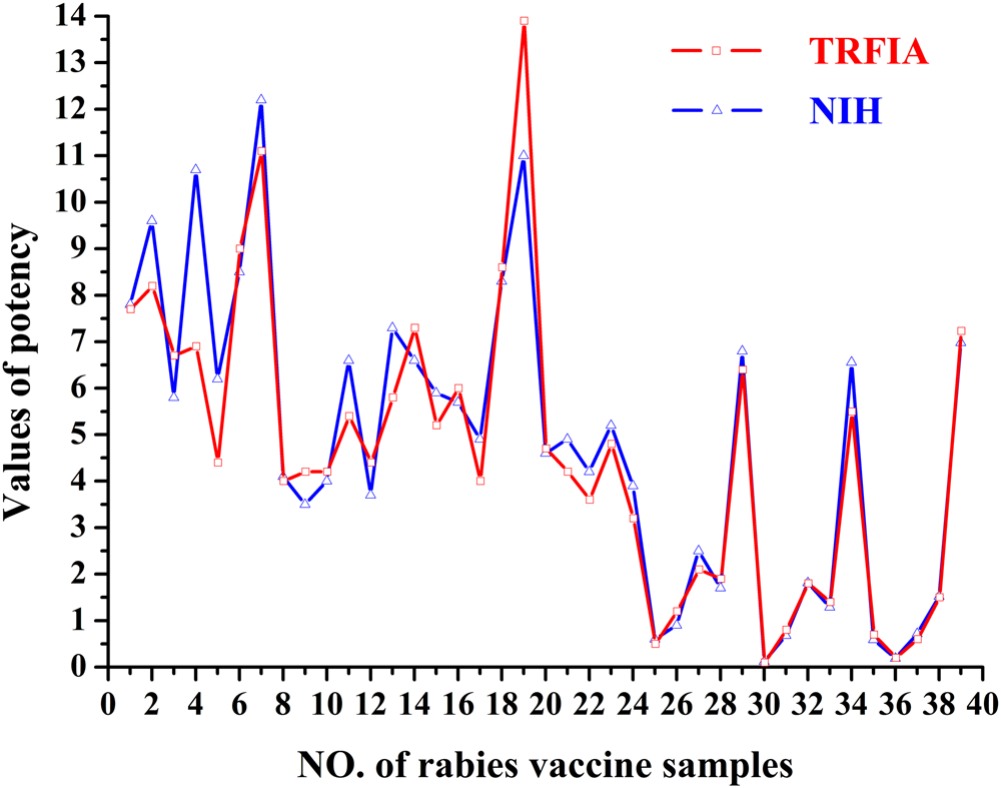
Result of TRFIA and NIH method for thirty nine rabies vaccine samples.

**Figure 6:**
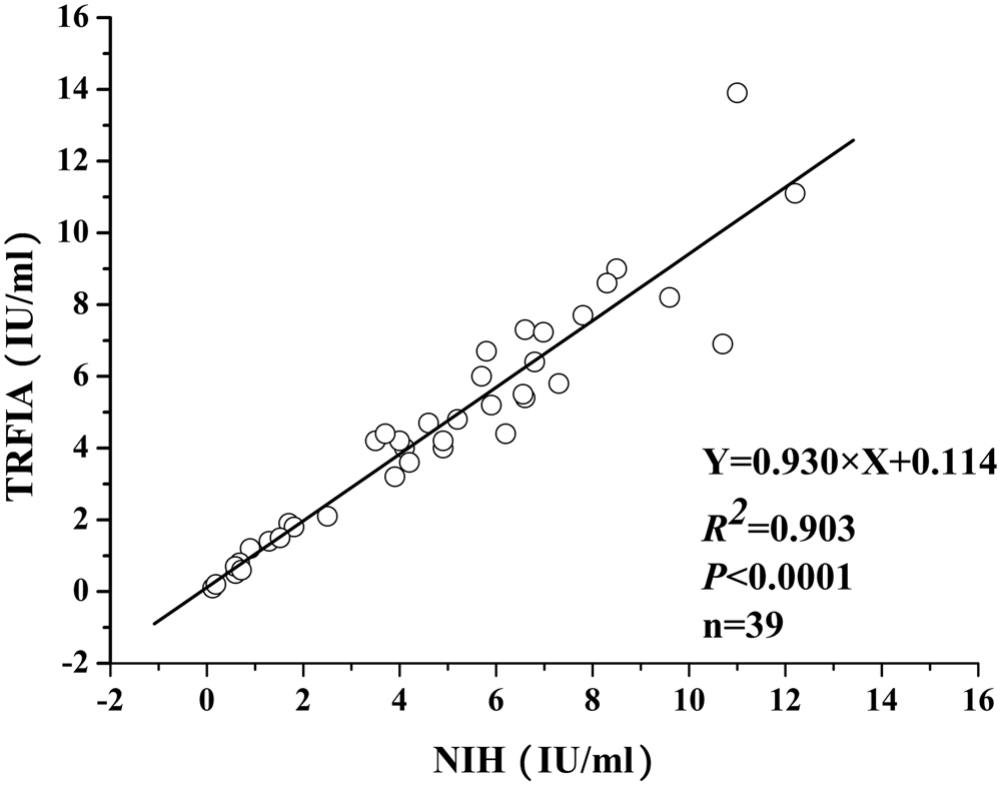
Graphical comparisons of the present TRFIA and NIH test results for evaluation of the potency of rabies vaccine samples.

Thirty nine vaccine samples were tested by the present TRFIA method and the potency of each sample was re-evaluated using the NIH test. As shown in Fig. 5, the variation tendency of results obtained from TRFIA method and NIH test was basically the same for those thirty nine vaccine samples. The comparisons of potency values obtained from these two methods were shown in Fig. 6. The correlation coefficient of potency values obtained from the present TRFIA method and NIH test was excellent, with a regression equation: ${\rm{Y}}=0.930\times {\rm{X}}+0.114$ (*r* ^2^ = 0.903, *P* < 0.0001).

## Discussion

In spite of all drawbacks, the NIH test still represents the “gold standard” against which any alternative method is judged^30^. However, NIH test’s own shortcomings determine that it is not suitable for large-scale use in the actual production process of rabies vaccine. In this case, vaccine manufacturers have to choose ELISA method for monitoring the quality control in each stage of vaccine production. As we know, ELISA cannot assay sample with very good performance and rapid detection. So the main aim of this work was to provide a more suitable *in vitro* method for the replacement of the *in vivo* potency test for rabies vaccine in the different stages of vaccine production process.

Fluorescence immunoassay, like other immunoassays involving non-isotopic labeling, has been well accepted as a stable, inexpensive, rapid, and sensitive method. However, conventional fluorescent labeling has a limited success in assay of analyte because of its high background, short decay time and broad spectrum, which make it difficult to be a qualified labeling for excellent quantitative analytical technique. Up to now, fluorescent lanthanide is a favorable choice owing to its excellent Stokes shift^31^. Its lifetime ranges 50–1000 μs (over four decades longer than the average background duration) depending on the temperature and the solvent presented^20^. These features can be utilized for optimization of the measurement conditions to get the maximal sensitivity and to minimize the signal spillover. As the application of TRFIA for quantification of rabies virus nucleoprotein in rabies vaccines was first reported by our research team^24^, TRFIA had been successfully introduced into the rabies vaccine field. As a successful replacement of ELISA, this nucleoprotein TRFIA was highly praised by the vaccine producers. Those preliminary research results had confirmed that TRFIA could be a reliable and interesting methodology in the field of rabies vaccine. Glycoprotein levels in a vaccine are often regarded as a surrogate for tests of vaccine potency. So we choose TRFIA method to be the suitable *in vitro* method for assaying rabies virus glycoprotein in rabies vaccine by using specific MAbs.

Monoclonal antibodies against this linear epitope of nucleoprotein have the potential to recognize native rabies virus nucleoprotein^32^, and expression of the nucleoprotein gene of rabies virus can be used as immune antigen and diagnostic reagent^24, 33^. So the preparation of nucleoprotein MAb will be carried out very smoothly. But unlike nucleoprotein MAb, specific glycoprotein MAbs used in this TRFIA have to recognize glycoprotein molecules folded in the native form, and without detecting the glycoprotein monomeric forms (such as soluble glycoprotein) which are poor immunogens^34^. Our research team spent a lot of work on the preparation of those specific glycoprotein MAbs in our laboratory. Fortunately, paired anti-glycoprotein MAbs (S053 and S036) were successfully screened out. Correlation coefficient (*r* ^2^ = 0.912) for TRFIA and NIH test results indicated the high specificity of S053 and S036 MAbs. These preliminary results supported the hypothesis that *in vivo* immunogenicity may be predicted from the *in vitro* assay of glycoprotein using the specific glycoprotein MAb.

We have described the establishment and preliminary validation of this novel TRFIA for the assay of rabies virus glycoprotein in rabies vaccines, and the method demonstrated satisfactory results when applied in practical samples. The minimum detectable dose of the present TRFIA was as low as 0.098 IU/ml. Thus, those samples with lower values could easily be detected by the present TRFIA. Samples with high values could be detected without dilution based on the detection range can be up to 500 IU/ml. The measurement of parameters was acceptable compared with other TRFIA^23, 27, 35, 36^, and better than most conventional ELISA^27^. Correlation coefficient (*r* ^2^ = 0.902) suggested that TRFIA and ELISA results showed good correlation. Due to its wider detection range and shorter reaction time, the present TRFIA can greatly save the operation time and workload for quality control in each stage of vaccine production. Base on the excellent measurement of parameters of the present TRFIA, we claimed that the present TRFIA could replace ELISA with credible results and rapid detection. It could perform as well as NIH test when applied to evaluation of the potency of rabies vaccine samples. What is more, this method demonstrated high sensitivity, wider effective detection range and excellent reproducibility for the assay of rabies virus glycoprotein, and offered additional benefit for rapid detection, resulting in a substantially faster assay. At present, it is a feasible and more suitable tool for the quality control in the process of rabies vaccine production, such as monitoring the production consistency of the rabies vaccines prior to their release to the market. We have reasons to believe that the present TRFIA should be a new and interesting methodology for the rabies vaccines field of potency testing. Based on this research, we established a good foundation for further development of the dual-label time-resolved fluoroimmunoassay for rabies glycoprotein and nucleoprotein by using the same platform as we did in our past research^23, 25^. Direct labeling of immune reagents with lanthanide chelates and lack of overlapping between Eu^3+^ and Sm^3+^ chelates allow to save more time and workload.
